# CT imaging of primary pancreatic lymphoma: experience from three referral centres for pancreatic diseases

**DOI:** 10.1007/s13244-017-0585-y

**Published:** 2018-01-15

**Authors:** Enrico Boninsegna, Giulia A. Zamboni, Davide Facchinelli, Charikleia Triantopoulou, Sofia Gourtsoyianni, Maria Chiara Ambrosetti, Dino Veneri, Achille Ambrosetti, Roberto Pozzi Mucelli

**Affiliations:** 1 Department of Radiology, Policlinico G. B. Rossi, University of Verona, Verona, Italy; 2 Department of Medicine, Section of Hematology, University of Verona, Verona, Italy; 3 Department of Radiology, Konstantopouleio General Hospital, Athens, Greece; 4 Department of Radiology, Guy’s and St Thomas’ NHS Foundation Trust, London, UK

**Keywords:** Pancreas, Lymphoma, Imaging, Computed tomography, Differential diagnosis

## Abstract

**Purpose:**

To describe CT characteristics of primary pancreatic lymphoma (PPL), a rare disease with features in common with adenocarcinoma.

**Materials and methods:**

Fourteen patients were enrolled. CT: unenhanced scan, contrast-enhanced pancreatic and venous phases. Image analysis: tumour location; peri-pancreatic vessel encasement; necrosis; enlarged lymph nodes; fat stranding; enlarged bile duct and pancreatic duct; neoplasm longest dimension, volume and density.

**Results:**

Histopathological diagnoses: follicular non-Hodgkin lymphoma (5/14), diffuse large B-cell lymphoma (6/14) and high-grade B-cell lymphoma not otherwise specified (3/14). Six of 14 PPLs were located in the pancreatic head and 7/14 in the body-tail; 1/14 involved the whole gland. In 5/14 cases the superior mesenteric artery and vein were encased; splenic vein and artery encasement was depicted in 2 PPLs. Necrosis was present in 2/14. Enlarged retroperitoneal lymph nodes were found in 11 cases and fat stranding in all patients. The bile duct was dilated in six cases and the pancreatic duct in five. Mean neoplasm longest diameter and volume were 8.05 cm and 210.8 cm^3^. Mean tumour attenuation values were 39.1 HU at baseline, 60.6 HU in the pancreatic phase and 71.4 HU in the venous phase.

**Conclusions:**

PPL presents as a large mass lesion with delayed homogeneous enhancement; peri-pancreatic fat stranding and vessel encasement are present, without vascular infiltration. Pancreatic duct dilatation is rare.

**Key points:**

• *Primary pancreatic lymphoma (PPL) is a rare haematological disease*

*• PPL presents imaging features in common with pancreatic carcinoma but also some distinctive findings*

•* The majority of PPLs are large lesions with delayed homogeneous enhancement*

•* Peri-pancreatic fat stranding and vessel encasement are common in PPL*

•* Vascular infiltration and pancreatic duct dilatation are rare in PPL*

## Introduction

Primary pancreatic lymphoma (PPL) is a very rare disease, representing only 0.1% of malignant lymphomas and 0.2% of primary pancreatic tumours [1, 2]. The World Health Organisation provides precise diagnostic criteria: the bulk of the disease is localised in the pancreas; adjacent lymph node involvement and distant spread may exist but the primary clinical presentation has to involve the pancreatic gland [3]. The disease can develop at any age, but usually affects elderly patients, with higher incidence in males. Clinical presentation is variable: in most cases the first manifestation is abdominal pain; other clinical findings include systemic symptoms such as fever, night sweats and weight loss. Jaundice and/or gastric or duodenal obstruction may be present [2]. These heterogeneous symptoms may overlap those of other pancreatic tumours, resulting in diagnostic difficulties [4, 5]. Histopathological examinations reveal a framework compatible with non-Hodgkin lymphoma, usually diffuse large B cell lymphoma (DLBCL). Nevertheless, other histological types have been reported including indolent diseases such as follicular and marginal zone lymphoma [3–6]. Diagnosis can be obtained through a percutaneous/endoscopic US-guided biopsy or exploratory laparotomy biopsy. The correct diagnosis is essential because, unlike epithelial tumours, patients with lymphoma do not benefit from surgery but from chemotherapy and indeed surgery may increase morbidity [5].

Up to now, reports of the CT imaging appearance of PPL are limited to a few papers including mainly case reports [7–10]. The aim of this study is to describe the imaging characteristics of PPL at CT based on a relatively large series of cases examined in three institutions in three different countries.

## Materials and methods

### Patient population

The Institutional Review Board (IRB) of the coordinating centre approved the research proposal. The IRB stated that this retrospective study was in compliance with the 1964 Helsinki Declaration and its later amendments, and the requirement for informed patient consent was waived. All patients with a histopathological diagnosis of PPL observed from January 2010 to July 2017 in three different institutes, all referral centres for pancreatic diseases (namely, centre A, coordinating centre; centre B; centre C) were evaluated. Patients were identified searching the medical record databases of radiology, pathology and surgery. Inclusion criteria were: the availability, for each patient, of one contrast-enhanced CT examination performed in one of the centres before the start of chemotherapy and a histopathological confirmation of the diagnosis. We excluded patients affected by a lymphoma located in other organs with secondary pancreatic involvement.

Thus, our study population consisted of 14 Caucasian patients: 7 examined in centre A, 5 in centre B and 2 in centre C. The population included eight females and six males, with a mean age of 62 years. The following clinical manifestations were present: abdominal pain or discomfort (14/14), jaundice (8/14), palpable mass (5/14) and systemic symptoms (fever, night sweats and weight loss, 7/14 patients). Pre-treatment laboratory tests demonstrated elevated serum lactate dehydrogenase (LDH) levels in four patients (> 300 mU/ml). The carbohydrate antigen 19–9 (CA19–9) serum level was normal in 14 cases and only slightly increased in 1 patient (27.15 U/ml, normal value < 25 U/ml).

Clinical characteristics of the current study population are summarised in Table 1.

**Table 1:** Clinical characteristics of patients affected by primary pancreatic lymphomas (PPLs)

Parameters	Number of patients (*n* = 14)
Age	
• Mean	62 years
• Range	41–85 years
Gender	
• Male	6 (42.9%)
• Female	8 (57.1%)
Symptoms	
• Abdominal pain or discomfort	14 (100%)
• Jaundice	8 (57.1%)
• Palpable mass	5 (35.7%)
• Systemic symptoms	7 (50.0%)

Histopathological diagnosis was reached with biopsy of the involved pancreas (US-guided percutaneous procedure: 6/14; US-guided endoscopic: 7/14) or endoscopic biopsy of the neoplasm involving the duodenum wall (1/14). All patients were treated with polychemotherapy associated with immunotherapy and CD-20 antibodies.

### Contrast-enhanced CT protocol

All CT examinations were performed with the same scanner type (a 64-row multidetector CT) in the three involved centres using the same imaging protocol. Triphasic CT was performed with patients in the supine position with the arms extended above the head. The scans included: unenhanced scan from the dome of the liver to the lower level of the pancreas; contrast-enhanced pancreatic parenchymal phase using the bolus-tracking technique (trigger threshold: 200 HU, scan delay after trigger: 15 s) with the same field of view; portal phase (70 s) from the liver dome to the symphysis pubis. All patients received 1.5 ml per kilogram of body weight intravenous iodinated contrast agent (Ultravist 370; Bayer Schering Pharma, Leverkusen, Germany) at an injection rate of 4 ml/s using an automatic injector through an antecubital vein and followed by 40–50 ml of saline solution chase at the same flow rate. Other scanning parameters were: tube voltage 120 kVp; automatic tube current modulation and slice thickness 2 mm.

### Image analysis

The imaging data obtained from MDCT were processed on commercially available workstations. In each participating centre one radiologist (radiologist A, with 12 years of experience in abdominal imaging; radiologist B, 13 years of experience; radiologist C, 12 years of experience) analysed the images from their own institution. A fourth radiologist from the coordinating centre, with 5 years of experience in abdominal imaging, assessed the data collected by the other radiologists to avoid inhomogeneities in the analyses. *Qualitative analysis* included: tumour location (head, body-tail or the whole pancreatic gland); presence of major peri-pancreatic vessel encasement (defined as circumferential involvement of the vessel [11]), in particular the caeliac axis, superior mesenteric artery, common hepatic artery, gastroduodenal artery, portal vein, superior mesenteric vein and splenic vein; presence of necrosis within the tumour (regions with attenuation between 10 and 30 HU in the unenhanced scan, without contrast enhancement [12]); presence of enlarged abdominal lymph nodes (shot axis > 10 mm); presence of fat stranding in the peri-pancreatic region (defined as abnormally increased attenuation in the fat, suggestive of infiltration of the mesenteric lymphatic vessels [13]); presence of an enlarged common bile duct (calibre > 10 mm); presence of an enlarged main pancreatic duct upstream of the neoplasm (calibre > 4 mm in the pancreatic head or >3 in the body-tail [14]).

The assessed *quantitative parameters* were: neoplasm longest dimension; volume of the lesion (calculated with the ellipsoid formula: 3 main diameters × 0.52); tumour density in Hounsfield units (non-contrast, pancreatic and venous phase).

### Statistical analysis

Categorical variables are presented as numbers and percentages. The distribution of continuous variables is reported as mean values and standard deviations. Statistical analyses were performed using MedCalc software for Windows, version 11.2.1.

## Results

Histopathological results were: follicular non-Hodgkin lymphoma in five patients, diffuse large B-cell lymphoma (DLBCL) in six patients and high-grade B-cell lymphoma not otherwise specified in three patients. The replicative Ki67 index score was below 25% in two cases, between 25% and 50% in four cases, between 50% and 75% in one patient and more than 75% in seven cases. The histopathological features are summarised in Table 2.

**Table 2:** Histopathological results

Histotype	Number of patients (*n* = 14)
• Follicular non-Hodgkin lymphoma	5
• Diffuse large B-cell lymphoma (DLBCL)	6
• High-grade B-cell lymphoma not otherwise specified	3
Replicative Ki67 index score	
• Ki67 ≤ 25%	2
• 25% < Ki67 ≤ 50%	4
• 50% < Ki67 ≤ 75%	1
• 75% < Ki67 ≤ 100%	7

**Fig. 1:**
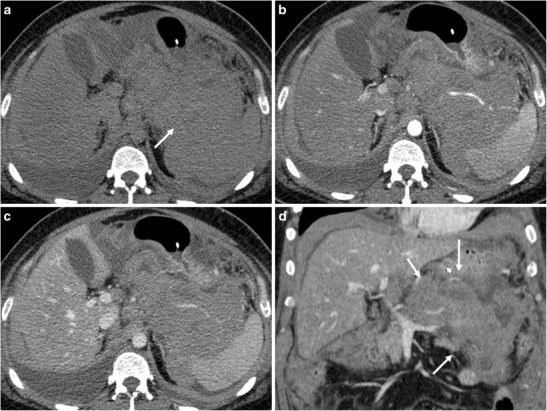
A 42-year-old male patient with primary pancreatic lymphoma, histotype diffuse large B-cell lymphoma (Ki 67 score > 95%). **a.** CT scan of the abdomen shows the presence of a large solid mass involving the pancreatic body and tail (arrow); the lesion reaches the spleen and almost encases it. **b, c** CT images after contrast medium administration in the arterial-pancreatic phase (**a**) and portal-venous phase (**b**): the neoplasm is hypodense, with progressive contrast enhancement. **d.** This coronal CT reconstruction allows better appreciation of the size and extension of the lesion (arrows)

**Fig. 2:**
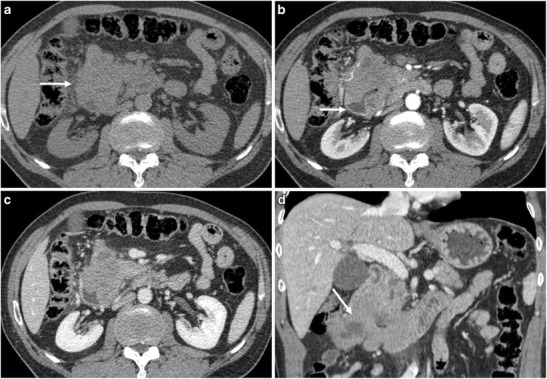
A 62-year-old male patient with primary pancreatic lymphoma, histotype high-grade B-cell lymphoma not otherwise specified (Ki 67 score > 90%). **a** CT scan of the abdomen shows the presence of a solid mass in the pancreatic head (arrow). **b, c, d** CT images after contrast medium administration in the arterial-pancreatic phase (**a**) and portal-venous phase (**b, c**): the neoplasm protrudes in the duodenum (arrow), as better demonstrated on coronal reconstruction (**d**). The histopathological diagnosis was reached with endoscopic biopsy of the involved duodenal wall

*Qualitative analysis.* Six out of 14 PPLs were located in the pancreatic head and 7 in the body-tail and 1 involved the whole gland (Figs. 1 and 2). In 5/14 cases the caeliac axis, superior mesenteric artery and superior mesenteric vein were encased; splenic vein and artery encasement was depicted in two PPLs. Even when the vessels were encased, there were no signs of infiltration, with no vessel wall irregularity or stenoses.

Two patients had tumoral necrosis, involving respectively about 20% and 50% of the pancreatic mass lesion. Enlarged retroperitoneal or mesenteric lymph nodes were depicted in 11/14 cases. Fat stranding in the peri-pancreatic region was observed in all patients. The common bile duct was dilated in 6/14 cases; the main pancreatic duct upstream of the neoplasm was enlarged in 5/14 patients. Qualitative parameters are summarised in Table 3.

**Table 3:** Results of the qualitative analysis

Parameters	Number of patients (*n* = 14)
Site	
• Head	6 (42.9%)
• Body/tail	7 (50.0%)
• Whole pancreas	1 (7.14%)
Major vessel encasement	
• Caeliac axis	5 (35.7%)
• Superior mesenteric artery	5 (35.7%)
• Superior mesenteric vein	5 (35.7%)
• Splenic artery	2 (14.3%)
• Splenic vein	2 (14.3%)
Presence of necrosis	2 (14.3%)
Enlarged lymph nodes	11 (78.6%)
Peri-pancreatic fat stranding	14 (100%)
Enlarged common hepatic duct	6 (42.9%)
Enlarged main pancreatic duct	5 (35.7%)

**Fig. 3:**
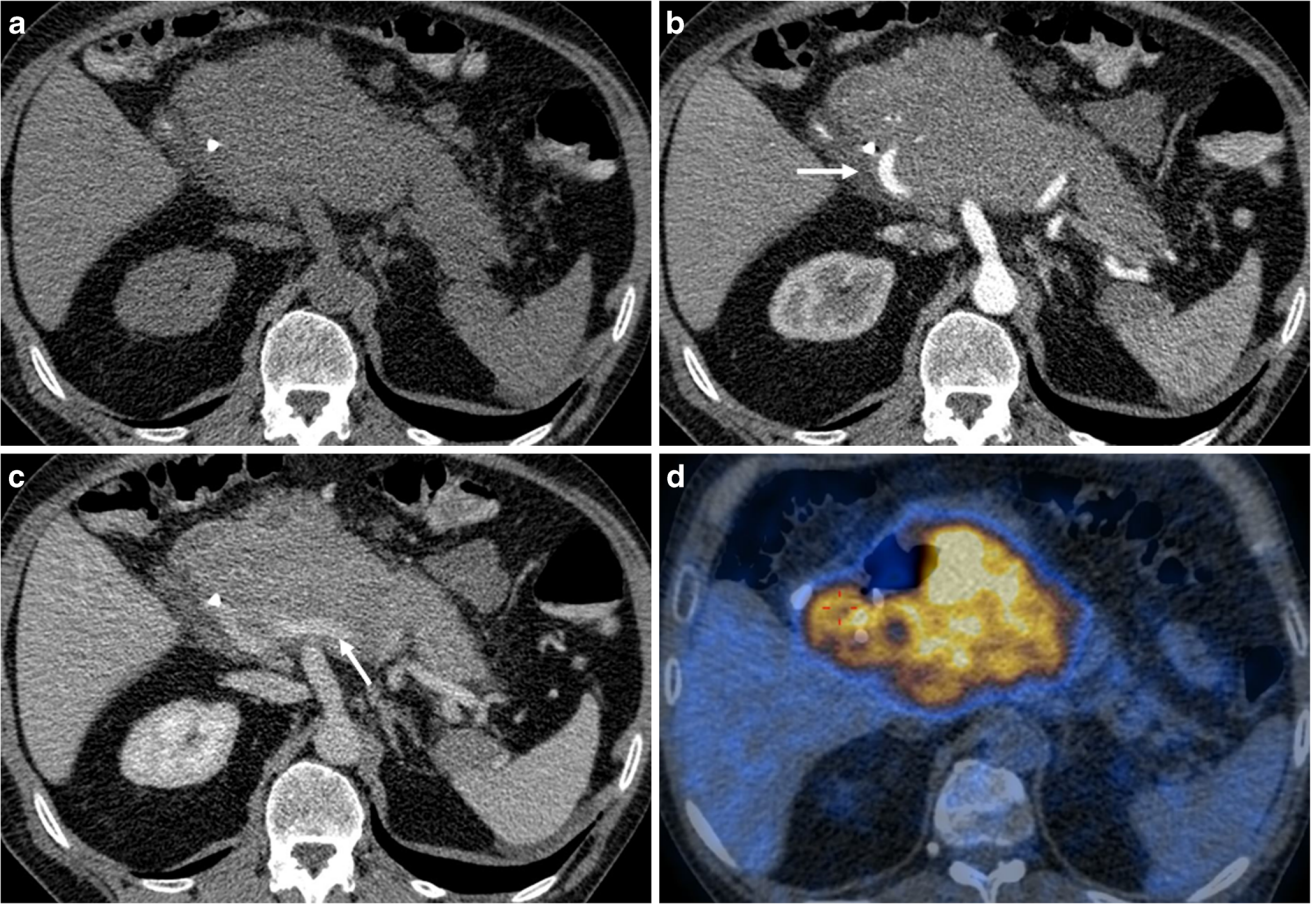
A 51-year-old male patient with primary pancreatic lymphoma, histotype diffuse large B-cell (Ki 67 score > 90%). **a** CT scan of the abdomen shows the presence of a large solid mass in the body-head of the pancreas. A biliary stent is seen. **b, c** CT images after contrast medium administration in the arterial-pancreatic phase (**a**) and portal-venous phase (**b**): the arteries and veins (arrows) are encased but not infiltrated. The main pancreatic duct is not enlarged. **d** This ^18^FDG PET-CT fusion image reveals high metabolic activity in the lesion

*Quantitative analysis.* Mean neoplasm longest dimension and volume were 8.05 cm and 210.8 cm^3^. Mean tumour attenuation values were 39.1 HU in the unenhanced scan, 60.6 HU in the pancreatic phase and 71.4 HU in the venous phase (Fig. 3). Quantitative parameters are summarised in Table 4.

**Table 4:** Results of the quantitative analysis

Parameters	Mean value ± standard deviation
• Longest dimension	8.05 ± 4.3 cm
• Volume	210.8 ± 308 cm^3^
• Attenuation value in the unenhanced scan	39.1 ± 5.9 HU
• Attenuation value in the arterial-pancreatic phase	60.6 ± 10 HU
• Attenuation value in the portal-venous phase	71.4 ± 8.7 HU

## Discussion

Although several case reports of PPLs have been published, there is still little information regarding the imaging features of these rare lesions [4, 5, 7, 8]. The current study encompasses 7 years of experience at three different institutions in three different countries and describes CT and histopathological features of 14 patients with PPL. The rarity of PPL makes it difficult to recognise this neoplasm since it presents characteristics in common with ductal adenocarcinoma, the most frequent pancreatic solid tumour or, more rarely, with hypovascular neuroendocrine tumours. PPL could also be confused with the focal or diffuse forms of autoimmune pancreatitis; all these conditions present different management approaches, with distinct treatment and prognosis [1, 7, 9].

Every patient in our series presented with abdominal pain or discomfort; other frequent clinical manifestations were jaundice, a palpable abdominal mass and systemic symptoms, such as fever, night sweats and weight loss. Unfortunately, pancreatic adenocarcinoma and autoimmune pancreatitis often have the same clinical picture [14]. As confirmed by the current study, the presence of an increased LDH serum level without an increased CA19–9 level favours a lymphomatous aetiology of the pancreatic mass [5]. The final histopathological diagnosis was available for every patient: the majority were histologically aggressive DLBCL or high-grade B-cell lymphoma not otherwise specified, with a high Ki67 index (> 75%). Only 5/14 presented low-grade malignancy (follicular lymphomas with Ki67 < 50%). According to the literature, the most common PPL histological subtype is DLBCL followed by follicular lymphoma. Before the 2016 WHO classification, most high-grade B-cell lymphomas not otherwise specified were included in the DLBCL group. Therefore, our data are in agreement with previous studies [5, 15].

**Fig. 4:**
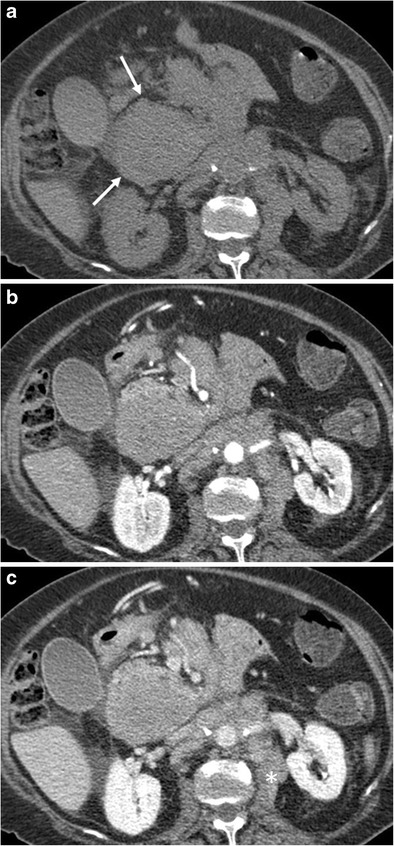
A 79-year-old female patient with primary pancreatic follicular lymphoma (Ki 67 score < 25%). **a** Unenhanced CT scan depicts a hypodense mass in the pancreatic head (arrows). **b, c** CT images after contrast medium administration in the arterial-pancreatic (**b**) and portal-venous phase (**c**): progressive enhancement of the neoplasm is visible. Enlarged retroperitoneal lymph nodes with ill-defined margins are depicted (*)

Some particular imaging features in patients with PPL were observed. There was not a predilection for a pancreatic region and the whole gland could be globally involved. Vascular encasement was typical, but significant irregularities in the vessels wall were never found (Fig. 3). This peculiarity could be helpful in the differential diagnosis with ductal adenocarcinoma, which causes vascular infiltration at early stages, with erosion of vessel walls, stenoses and eventually tumoral thrombosis [14]. The pancreatic parenchyma affected by lymphoma has low attenuation values compared to the healthy pancreas; as opposed to adenocarcinoma, areas of necrosis are rare. After contrast medium administration the attenuation is higher in the portal-venous phase compared to the arterial-pancreatic phase (Fig. 4). Enlarged retroperitoneal lymph nodes are frequent and fat stranding is always present. The double duct sign, simultaneous dilation of the common bile and pancreatic ducts, typical in patients with adenocarcinoma of the pancreatic head, was only depicted in 5/14 PPLs [1, 14–17].

In the most difficult cases other imaging modalities are recommended in addition to CT: MRI, in particular MR cholangiography sequences, can give important information about ductal system morphology (a single stricture of the pancreatic duct with an upstream enlarged duct is highly suggestive of ductal adenocarcinoma) [16]. Evaluation of stenoses in the ductal system could be improved by the administration of secretin to distinguish between the infiltrated main duct (negative duct-penetrating sign after drug administration, typical of adenocarcinoma) and compressed duct (positive duct-penetrating sign, suggestive of a lymphomatous process) [16]. Diffusion-weighted imaging helps to recognise the areas of pancreatic parenchyma involved by the neoplasm and could have a role in the evaluation of treatment response [17]. Nuclear medicine, in particular ^18^FDG PET-CT, is indicated to depict involved lymph nodes in the whole body and to assess the metabolic activity of the primary neoplasm. The recently proposed PERCIST system (PET Response Criteria in Solid Tumours) evaluates changes in the standardised uptake value (SUV) of ^18^FDG to assess treatment response [18].

Most of the above-mentioned imaging features could allow distinguishing PPL from pancreatic adenocarcinoma, but the differential diagnosis with autoimmune pancreatitis remains difficult. As reported by previous authors, the size of the parenchyma involved by the autoimmune process is smaller than for PPL [3]. In addition, symptoms of autoimmune pancreatitis are less severe and the disease is often associated with increased IgG4 serum levels [19]. According to our experience, it is mandatory to integrate imaging findings with the clinical and laboratory results to make the correct diagnostic hypothesis; histopathological confirmation is always necessary, also to evaluate the lymphoma subtype.

The present study has some limitations, namely, its retrospective design and the relatively small sample size. A larger prospective study would be required to corroborate our findings.

In conclusion, the most suggestive CT signs of primary pancreatic lymphoma are a large hypodense mass lesion with progressive contrast enhancement, absence of necrosis, lack of dilation of the main pancreatic duct and presence of vessel encasement without infiltration.
